# UBE2M Drives Hepatocellular Cancer Progression as a p53 Negative Regulator by Binding to MDM2 and Ribosomal Protein L11

**DOI:** 10.3390/cancers13194901

**Published:** 2021-09-29

**Authors:** Ju-Ha Kim, Ji Hoon Jung, Hyo-Jung Lee, Deok-Yong Sim, Eunji Im, Jieon Park, Woon-Yi Park, Chi-Hoon Ahn, Bum-Sang Shim, Bonglee Kim, Sung-Hoon Kim

**Affiliations:** Molecular Cancer Target Herbal Research Laboratory, College of Korean Medicine, Kyung Hee University, Seoul 02447, Korea; 964juha@khu.ac.kr (J.-H.K.); johnsperfume@khu.ac.kr (J.H.J.); hyonice77@khu.ac.kr (H.-J.L.); simdy0821@khu.ac.kr (D.-Y.S.); ji4137@khu.ac.kr (E.I.); jieon77@khu.ac.kr (J.P.); wy1319@khu.ac.kr (W.-Y.P.); ach2565@khu.ac.kr (C.-H.A.); eshimbs@khu.ac.kr (B.-S.S.)

**Keywords:** UBE2M, MDM2, p53, ribosomal protein L11, hepatocellular carcinoma

## Abstract

**Simple Summary:**

Herein, the oncogenic role of UBE2M as an E2 NEDD8-conjugating enzyme was explored in hepatocellular carcinoma (HCC) cells, since neddylation plays a critical role in tumorigenesis. To address this issue, human tissue array and TCGA analysis were conducted in HCCs to find overexpression of UBE2M in HCCs. In addition, a differential profile was confirmed in UBE2M-depleted HepG2 cells. Furthermore, UBE2M depletion activated p53 expression and stability, while the ectopic expression of UBE2M disturbed p53 activation and enhanced degradation of exogenous p53 mediated by MDM2 in HepG2 cells via binding to MDM2 and ribosomal protein L11 by immunoprecipitation and immunofluorescence. These findings provide evidence that UBE2M is critically involved in liver cancer progression as a p53 negative regulator by binding to MDM2 and ribosomal protein L11.

**Abstract:**

Though UBE2M, an E2 NEDD8-conjugating enzyme, is overexpressed in HepG2, Hep3B, Huh7 and PLC/PRF5 HCCs with poor prognosis by human tissue array and TCGA analysis, its underlying oncogenic mechanism remains unclear. Herein, UBE2M depletion suppressed viability and proliferation and induced cell cycle arrest and apoptosis via cleavages of PARP and caspase 3 and upregulation of p53, Bax and PUMA in HepG2, Huh7 and Hep3B cells. Furthermore, UBE2M depletion activated p53 expression and stability, while the ectopic expression of UBE2M disturbed p53 activation and enhanced degradation of exogenous p53 mediated by MDM2 in HepG2 cells. Interestingly, UBE2M binds to MDM2 or ribosomal protein L11, but not p53 in HepG2 cells, despite crosstalk between p53 and UBE2M. Consistently, the colocalization between UBE2M and MDM2 was observed by immunofluorescence. Notably, L11 was required in p53 activation by UBE2M depletion. Furthermore, UBE2M depletion retarded the growth of HepG2 cells in athymic nude mice along with elevated p53. Overall, these findings suggest that UBE2M promotes cancer progression as a p53 negative regulator by binding to MDM2 and ribosomal protein L11 in HCCs.

## 1. Introduction

Hepatocellular carcinoma (HCC) is known as the sixth most common cancer and the third leading cause of cancer-associated mortality in the world [1]. Recently, HCC has been associated with neddylation [2] and various signaling pathways [3] and is genetically and phenotypically regarded as a heterogeneous cancer [4].

Neddylation is considered as one of important signaling pathways in tumorigenesis, since post-translational modification critically modulates protein activation by ubiquitin-proteasome system (UPS) [5,6,7]. Thus, UPS dysregulation was found in multiple myeloma [8], uveal melanoma [9], lung cancer [10] and liver cancer [11]

It is well documented that neddylation cascades are activated by neural precursor cell-expressed, developmentally downregulated protein 8 (NEDD8) E1 activating enzyme (NAE) and NEDD8 E2 enzymes (UBE2M (UBC12) and UBE2F) and eventually conjugated by E3 enzymes, such as RBX1 and RBX2 [12,13]. Among neddylation cascade proteins, UBE2M is known as one of the neddylation ligase complexes, such as cullin-RING ligases (CRLs), RBX1 and ROC1, for poly-ubiquitin conjugation [14] and targets degradation of UBE2F [15,16,17] and p27 (Kip1) [18]. In addition, UBE2M acts as a stress-inducible dual gene for neddylation and ubiquitylation [19] and promotes proliferation and migration in HCCs via activation of β catenin and cyclin D1 [20], along with overexpression in several cancers, including HCCs [21], H1299 lung cancer [22] and osteosarcoma [23].

p53 is well known as a tumor suppressor [24,25,26] by inducing cell-cycle arrest and apoptosis in several cancers [27,28]. Accumulating evidence reveals that MDM2 ubiquitinates p53 as E3 ubiquitin ligase, since MDM2 binds to p53’s N-terminal site and blocks its transcriptional activity [29,30] through a negative feedback loop between them [31]. Furthermore, emerging evidence shows that ribosomal protein L11 for ribosome biogenesis regulates the MDM2–p53 signaling pathway [32,33]. Interestingly, Macias et al. claimed that inhibition of ribosomal biogenesis activates p53 via ribosomal protein-mediated suppression of MDM2 E3 ligase [34], while Sun et al. reported that ribosomal proteins L5, L11 and L23 activate p53 by reducing the MDM2–p53 feedback circuit [19].

Though UBE2M is known to act as an oncogene for neddylation and ubiquitylation and is associated with the activation of β catenin and cyclin D1 [20], the oncogenic mechanisms are not fully understood. Hence, in the present study, the underlying molecular mechanisms of UBE2M were explored in association with p53/MDM2 and ribosomal protein L11.

## 2. Materials and Methods

### 2.1. Cell Culture

Human hepatocellular carcinoma cell lines such as HepG2, Hep3B and Huh7, PLC/PRF5 cells and human colorectal cancer HCT116 cells were obtained from ATCC (Manassas, VA, USA). HepG2 cells were cultured in Modified Eagle Medium (MEM, catalog NO. LM 007-54, WelGENE, Gyeongsangbuk-do, Korea). Hep3B cells were cultured in Dulbecco’s Modified Eagle Medium (DMEM, catalog NO. LM 001-05, WelGENE, Gyeongsangbuk-do). Huh7, PLC/PRF5 and HCT116 cells were cultured in Roswell Park Memorial Institute 1640 (RPMI, catalog NO. LM 011-01, WelGENE). All cells were cultured in the aforementioned medium supplemented with 10% heat-inactivated fatal bovine serum (FBS, WelGENE) and 1% antibiotic solution (100 units/mL penicillin and 100 µg/mL streptomycin, WelGENE) at 37 °C 5% CO_2_.

### 2.2. Tissue Microarray and Immunohistochemistry

HCC patient tissue microarray plates with 80 cases of hepatocellular carcinoma were purchased from US Biomax (HLivH160CS01, MD, USA) for immunohistochemistry (IHC) staining with Discovery XT (Roche, Basel, Discovery XT (Roche, Basel, Switzerland ). Each plate includes tumor and matched normal adjacent tumor tissues. The tissues were fixed with 4% paraformaldehyde, dehydrated, embedded in paraffin and sectioned at 4 µm. Sections were deparaffinized, rehydrated and incubated with 3% H_2_O_2_. After antigen repair and being blocked, the slides were incubated with mouse monoclonal antibody against UBE2M (1:200) (Cat.No. 109507, Abcam, Waltham, MA, USA) and p53 (Cat. No. sc-126, Santacruz, Rio Grande, TX, USA) at 4 °C overnight. Subsequently, the slides were incubated with the secondary antibody at room temperature for 30 minutes and then incubated with streptavidin peroxidase complex. Staining was performed using a 3,3-diaminobenzidine (DAB) substrate kit for peroxidase reaction and counterstained with hematoxylin. Finally, the slides were analyzed with a light microscope.

### 2.3. RNA Interference and Plasmid Transfection

The cells were seeded onto culture plates overnight and transfected with the mixtures of p53 siRNA or UBE2M siRNA or negative control siRNA purchased from Bioneer (Daejeon, ROK) adjusted at 40 nM by using an INTERFERin transfection reagent (Polyplus, Illkirch, Illkirch, France) according to the manufacturer’s protocol. The transfected cells were incubated for 60–72 h for the next experiment. In addition, RGS/His-UBE2M, Flag-p53, MDM2 and pcDNA 3.0 plasmids, purchased from Addgene (Cambridge, MA, USA), were transfected into the cells by using the Turbofect transfection reagent (Thermo Fisher Scientific, Waltham, MA, USA) and then incubated for 24–48 h for further study. UBE2M siRNA-#1 (sense strand, CUG AUG AGG GCU UCU ACA A=tt and antisense strand, UUG UAG AAG CCC UCA UCA G=tt) and UBE2M siRNA-#2 (sense strand, GAA AUA GGG UUG GCG CAU A=tt and antisense strand, UAU GCG CCA ACC CUA UUU C=tt) were purchased from Bioneer (Daejeon, ROK).

### 2.4. Next Generation Sequence (NGS) Analysis

RNAs from UBE2M depleted HepG2 cells were isolated and the quality was checked by e-biogen corporation (Seoul, Korea). Samples were progressed using the QuantSeq 3’ mRNA-Seq service of the NGS sequence analysis (NextSeq 500, Illumina). mRNA expression profiling and analysis were performed by the EX-DEGA program (e-biogen). Clustering heat map analysis was performed by the MeV software (version 4.9.0). NGS raw data were deposited in the NCBI’s BioProject database (accession number, PRJNA722599).

### 2.5. Quantitative Reverse Transcription Polymerase Chain Reaction (qRT-PCR)

RNAs isolated from HepG2 cells transfected with siCTL and UBE2M siRNA were lysed by QIAZOL (Qiagen, Hilden, Germany) according to the manufacturer’s protocol. A total of 2 μg of the RNA samples was synthesized to complementary DNA with Oligo dT (Bioneer, Daejeon, ROK), dNTP (Takara, Shiga, Japan) and M-MLV reverse transcriptase (Enzynomics, Daejeon, Korea) following the manufacturer (Enzynomics)’s protocol. Primers were purchased synthesized by Bioneer (Daejeon, Korea). The primers for p53, Bax, PUMA, UBE2M and GAPDH were as follows: p53, 5′-AGGACAGGCACAACACGCACC-3′ and 5′-TAACAGTTCCTGCATGGGCGGC-3′; Bax, 5′-TGCCACTCGGAAAAAGACCT-3′ and 5′-CTGCAGAGGATGATTGCCG-3′; PUMA, 5′-CCTGGAGGGTCCTGTACAATCT-3′ and 5′-GCACCTAATTGGGCTCCATCT-3′; UBE2M, 5′-AGTTGAGGAGGTCGTCTG-3′ and 5′-AGAAGAAGGAGGAGGATC-3′; GAPDH, 5′-GACGGTGCCATGGAATTTGC-3′ and 5′-ATGGGGAAGGTGAAGGTCGG-3′.

### 2.6. Western Blotting

Cells transfected with siCTL and UBE2M siRNA were lysed in NP40 buffer containing 50 mM Tris/HCL (pH7.5), 0.5% NonidetP-40, 1 mM EDTA, 120 mM NaCl, 1 mM dithiothreitol, 0.2 mM phenylmethylsufonylfluoride with protease inhibitors cocktails (Roche, Basel, Switzeland) and phosphatase inhibitors (Merck kGaA, Darmstadt, Germany). The lysates were quantified by the DC Protein Assay Kit II (Bio-Rad, Hercules, CA, USA). The protein samples were electrophoresed on 8–15% SDS-polyacrylamide gels and transferred to nitrocellulose membranes. Membranes were blocked with TBST-diluted 5% skim milk for 1 h at room temperature or TBST-diluted 5% BSA for 4 h at 4 °C. Then they were incubated with primary antibodies of PARP (Cat. No. 9542, Cell Signaling Technology, Danvers, MA, USA), cleaved caspase-3 (Cat.No. 9664, Cell Signaling Technology), p53 (Cat. No. sc-126, Santacruz, Rio Grande, TX, USA), MDM2 (Cat. No. sc-965, Santacruz), RPL5 (Cat. No. 14568, Cell Signaling Technology), RPL11 (Cat. No. 16277-1-AP, Proteintech, Rosemeont, IL, USA), UBE2M (Cat. No. sc-390064, Santacruz) and β-actin (Cat. No. A2228, Merck KGaA) diluted in 5% BSA in TBST overnight at 4 °C, washed three times for 10 min with TBST and incubated with HRP-conjugated secondary antibodies (Cell Signaling Technology) for 2 h. Expression was visualized by using an ECL Immunoblotting detection reagent (GE Healthcare, Chicago, IL, USA). File S1. Uncropped western blots.

### 2.7. Fractionation of Nuclear and Cytoplasmic Extract

Nuclear extraction was conducted using an NE- PER Nuclear Cytoplasmic Extraction Reagent kit (Thermo Scientific, Waltham, MA, USA) according to the manufacturer’s instructions. In brief, the transfected HepG2 cell pellets were suspended in cytoplasmic extraction reagent I by vortexing and incubated on ice for 10 min; then, a second cytoplasmic extraction reagent II was added. After the cells were centrifuged, the supernatant fraction (cytoplasmic extract) was transferred to a prechilled tube. The insoluble pellet fraction containing crude nuclei was resuspended in a nuclear extraction reagent. The final supernatant, constituting the nuclear extract, was used for the subsequent experiments.

### 2.8. Cycloheximide Assay

HepG2 cells transfected with siCTL and UBE2M siRNA for 72 h were exposed to 50 μg/mL of cycloheximide (CHX, Merck KGaA, Darmstadt, Germany) for the indicated concentrations and time points and Western blotting was performed [35].

### 2.9. Immunoprecipitation

HepG2 cells transfected with siCTL and UBE2M siRNA in absence or presence of MG132 (Merck KGaA) were lysed according to Western blotting protocols and quantitated. A total of 2 μg of antibodies for UBE2M (Cat. No. sc-390064, Santacruz) and MDM2 (Cat. No. sc-965, Santacruz) or ribosomal protein 11 or p53 was added to 500 μg of lysate and incubated at 4 °C in the rotator overnight. A volume of 30 μL of Protein G beads (Santacruz) was added and rotated at 4 °C for 4 h. Lysates were washed three times with a lysis buffer. The bound proteins were immunoblotted as indicated above. Protein amounts of input were 10% of immunoprecipitated samples.

### 2.10. Ubiquitylation Assay

Hep3B or H1299 cells transfected with siRNAs (siCTL or UBE2M siRNA) and plasmids (pcDNA3.0, Flag-p53, MDM2, HA-Ub and/or RGS/His-UBE2M) following addition of 20 μM proteasome inhibitor MG132 for 2 h in H1299 p53 mutant cells were lysed and immunoprecipitated with anti-HA antibody and protein G-agarose beads and immunoblotted with anti-Flag, UBE2M and β-actin antibody.

### 2.11. Immunofluorescence

HepG2 cells transfected with control or UBE2M siRNA were fixed with 4% paraformaldehyde for 20 min at room temperature and permeabilized with 0.1% Triton X-100 for 2 min on ice. The cells were labeled with primary antibodies of UBE2M and MDM2 diluted in 1% BSA/PBS overnight at 4 °C; then, they were exposed to secondary Alexa fluor (Invitrogen) diluted with 1% BSA/PBS for 2 h at room temperature. The samples were mounted with mounting medium containing DAPI and were visualized using an Olympus LUOVIEWFV10i (Olympus, Tokyo, Japan) confocal microscope and Delta Vision imaging system.

### 2.12. Establishment of UBE2M shRNA HCC Cell Lines

To establish HepG2 cell lines stably expressing UBE2M shRNA, UBE2M shRNA recombinant vectors and transfection mixtures (Turbofect, Thermo Fisher Scientific) were transfected into HepG2 cells. The transfected cells were grown in the medium supplemented with puromycin at 4 μg/mL for approximately 14 days to eliminate the untransfected cells. Then, the macroscopic clones were picked out and continuously passaged in the medium supplemented with puromycin (0.5 ng/mL–1.5 ng/mL). UBE2M protein expression was checked in HepG2 cells transfected with control shRNA and UBE2M shRNA by Western blotting for the following animal study.

### 2.13. In Vivo Xenograft Model

According to Animal Use Protocol (IACU number: KHUASP-19-208) approved by Kyung Hee University IACU Committee, an animal study was performed. A total of 10 Balb/c male athymic nude mice were randomly assigned to two groups (5 mice per group). UBE2M shRNA HepG2 cells or intact HepG2 cells were subcutaneously injected into the flank of Balb/c male athymic nude mouse (5 weeks old; Narabio, Korea) at the concentration of 5 × 10^6^ cells/200 μL. Tumor size was monitored for 39 days. All mice were sacrificed on day 39 after implantation and necropsy was carried out. In addition, IHC and Western blotting were conducted with tumors isolated from the mice.

### 2.14. Statistical Analysis

Data are expressed as means ± SD from at least three independent experiments. A Student’s *t*-test for two-group comparison and a one way analysis of variance (ANOVA) followed by a Tukey’s post-hoc test were conducted for multi-group comparison using the GraphPad Prism software (Version 5.0, San Diego, CA, USA). Significant differences were considered if the *p* value was less than 0.05.

## 3. Results

### 3.1. UBE2M Is Overexpressed in HCCs with Poor Prognosis: Its Depletion Exerts Antiproliferative and Apoptotic Effect in HCCs

**Figure 1:**
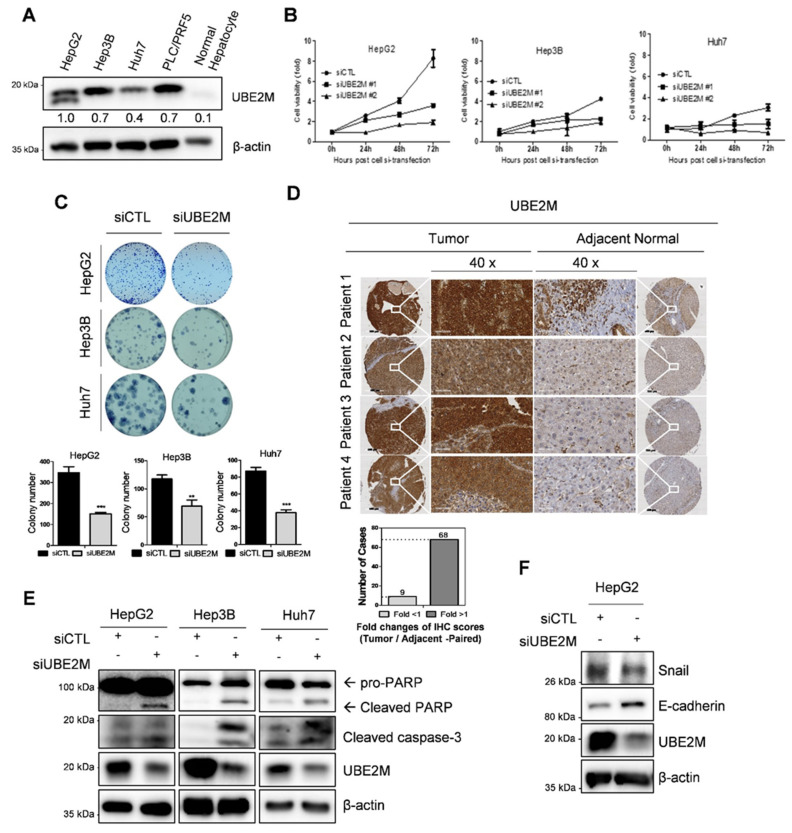
UBE2M overexpression in HCC cell lines and patient tissues, and cytotoxic and anti-proliferative effect of UBE2M depletion. (**A**) Endogenous expression level of UBE2M in hepatocellular carcinoma cell lines by Western blotting. (**B**) Effect of UBE2M depletion on the viability of HepG2, Hep3B and Huh7 cells. Cells were transfected with control and/or UBE2M siRNA-#1/siRNA-#2 and its cell viability was evaluated by MTT assay. Data represent means ± S.D from three independent experiments. (**C**) Effect of UBE2M depletion on the number of colonies in HepG2, Hep3B and Huh7 cells transfected with control and/or siRNA. Cells were cultured for 2 weeks in 12 well culture plate and stained. The number of colonies was counted. (**D**) UBE2M expression level in 80 patients’ liver cancer tissues and paired adjacent tissues using immunohistochemistry. 40× magnification. Data represent means ± S.D from three independent experiments. ** *p* < 0.01, *** *p* < 0.001 vs. untreated control. (**E**) Effect of UBE2M depletion on PARP cleavage in HepG2, Hep3B and Huh7 cells transfected with control and/or UBE2M siRNA. Cells were lysed and immunoblotted with antibodies of PARP, cleaved caspase-3, UBE2M and β-actin. (**F**) Effect of UBE2M depletion on Snail, E-cadherin in HepG2 cells.

UBE2M was overexpressed in human hepatocellular carcinoma (HCC), such as HepG2, Hep3B, Huh7 cells and PLC/PRF5 cells, but not in normal hepatocytes by Western blotting (Figure 1A) and tissues array (Figure 1D). One HepG2 cell line is *tp53* WT cell type and the other cell line is *tp53* deletion (Hep3B) and mutant types (Huh7 and PLC/PRF5). TCGA also revealed UBE2M was overexpressed at mRNA level in HCCs with poor survival rates (Figure S1A,B). In addition, UBE2M depletion reduced viability (Figure 1B) and the number of colonies for long term proliferation (Figure 1C) and induced cell-cycle arrest (data not shown) in HepG2, Hep3B and Huh7 cells. Transfection efficiency was confirmed in a time course transfection in HepG2 cells transfected by UBE2M siRNA plasmid by Western blotting (Figure S1A). Furthermore, UBE2M knockdown induced cleavages of PARP and caspase 3 in HepG2, Hep3B and Huh7 cells (Figure 1E), attenuated the expression of Snail and activated E-cadherin in HeG2 cells (Figure 1F).

### 3.2. p53/MDM2 and RPL Related Genes Were More Associated in UBE2M-Depleted HCCs

**Figure 2:**
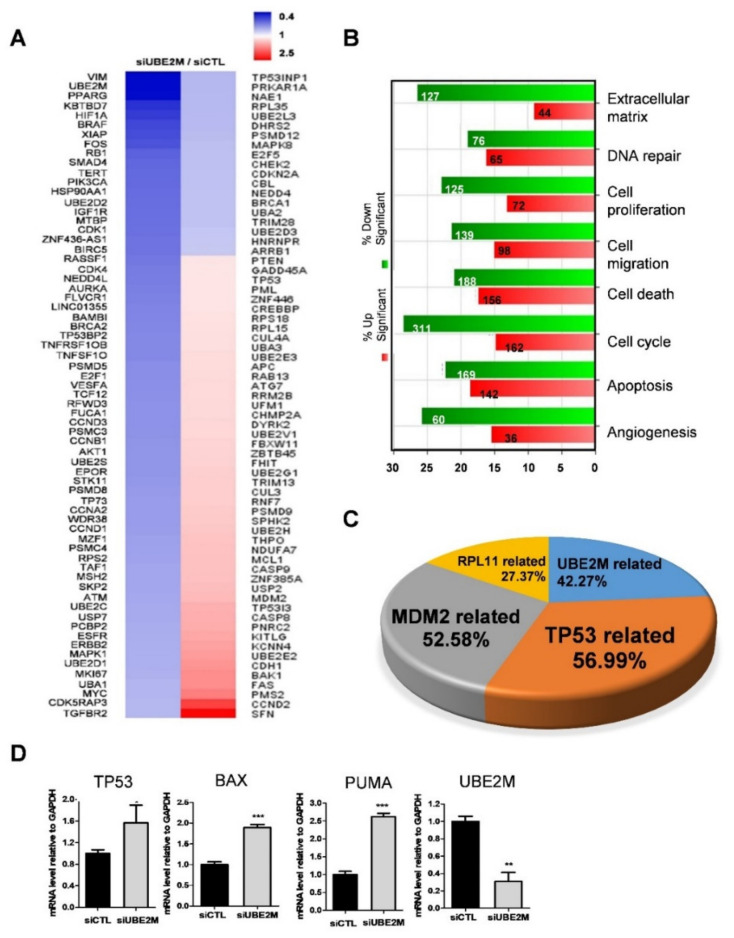
Differentially expressed gene profile is mainly associated with p53-related signaling and UBE2M depletion induces cell-cycle arrest and apoptosis in HCCs. (**A**) Heat map of genes enriched in UBE2M-depleted HepG2 cells. Blue and red represent increased and decreased expression of genes, respectively. (**B**) Gene ontology analysis for related signaling pathways. Green and red represent increased and decreased expression of genes, respectively. (**C**) Gene analysis for TP53-, UBE2M-, RPL11-, MDM2-related genes in a pie chart. (**D**) Effect of UBE2M depletion on TP53, Bax and PUMA in HepG2 cells by qRT-PCR. RNAs isolated from HepG2 cells transfected with control and/or UBE2M siRNA were lysed and subjected to qRT-PCR. * *p* < 0.05, ** < 0.01, *** *p* < 0.001 vs. untreated control.

**Figure 3:**
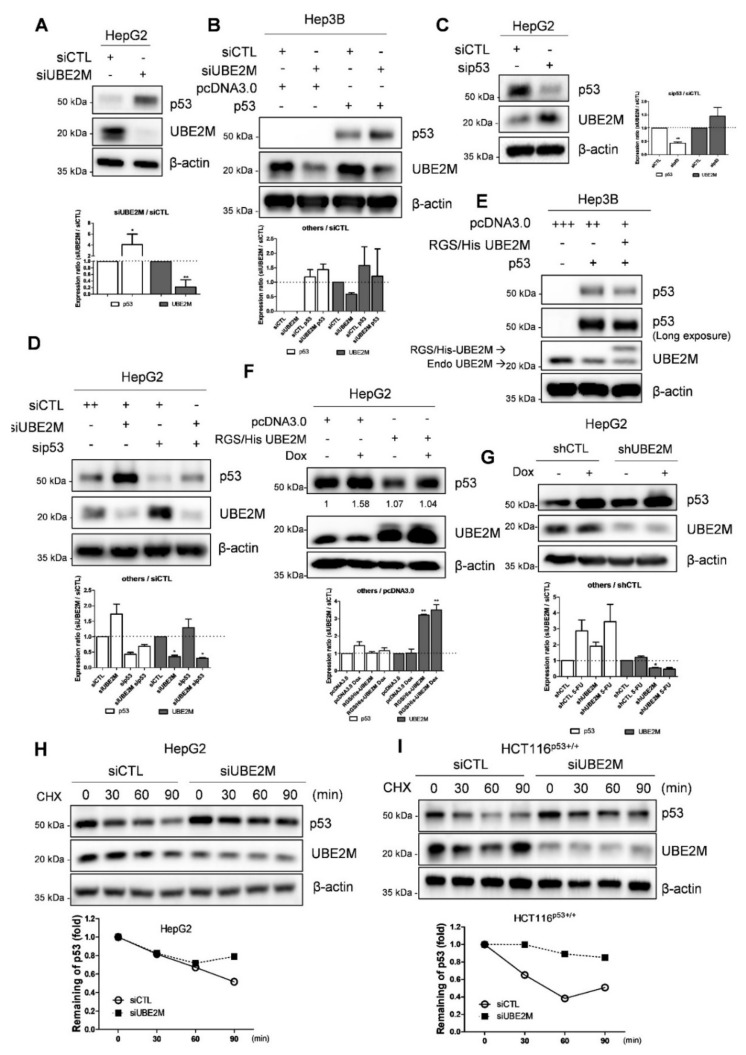
UBE2M depletion activates p53 and maintains its stability in HCCs. (**A**) Effect of UBE2M depletion on p53 in HepG2 cells. HepG2 cells were transfected with control siRNA and UBE2M siRNA or p53 siRNA for 72 h and were subjected to Western blotting with antibodies of p53, UBE2M and β-actin. (**B**) Effect of UBE2M depletion on p53 in Hep3B cells. Hep3B cells were transfected by control siRNA, UBE2M siRNA, pcDNA3.0 and p53 plasmids and were subjected to Western blotting with antibodies of p53, UBE2M and β-actin. (**C**) Effect of p53 depletion on UBE2M in HepG2 cells. (**D**) Effect of UBE2M depletion and/or p53 knockdown on p53 in HepG2 cells. (**E**) Effect of UBE2M overexpression on p53 in Hep3B cells. Hep3B cells were transfected with pcDNA3.0 RGS/His UBE2M and p53 plasmids and then were subjected to Western blotting with antibodies of p53, UBE2M and β-actin. (**F**) Effect of UBE2M overexpression on p53 in HepG2 cells exposed to doxorubicin. HepG2 cells were transfected with control siRNA, UBE2M siRNA, pcDNA3.0 and p53 plasmids with or without p53 activator Doxorubicin (0.1 μM) treatment and then were subjected to Western blotting with antibodies of p53, UBE2M and β-actin. (**G**) Effect of UBE2M depletion on p53 in HepG2 cells exposed to doxorubicin. (**H**) Effect of UBE2M overexpression on p53 stability in HepG2 cells in the presence of cycloheximide. HepG2 cells transfected with control siRNA and RGS/His UBE2M for 72 h were treated with cycloheximide (CHX) for 30, 60 and 90 min and were subjected to Western blotting with antibodies of p53, UBE2M and β-actin. Three independent assays were conducted in triplicate. (**I**) Effect of UBE2M depletion on p53 stability in HCT116p53+/+ cells in the presence of cycloheximide. HCT116p53+/+ cells transfected with control and UBE2M shRNA for 72 h were treated with CHX for 30, 60 and 90 min before harvesting cells and were subjected to Western blotting with antibodies of p53, UBE2M and β-actin. Three independent assays were conducted in triplicate. * *p* < 0.05 vs. siCTL. ** *p* < 0.01 vs. pcDNA3.0.

An NGS sequence analysis was conducted in UBE2M-depleted HepG2 cells. Herein, several genes were differentially expressed with upregulation (red) or downregulation (blue) (Figure 2A). In addition, the gene ontology analysis classified affected genes and signaling pathways into extracellular matrix, DNA repair, cell proliferation, cell migration, cell cycle, apoptosis and angiogenesis (Figure 2B). Of note, TP53-related genes (56.99%), MDM2-related genes (52.68%) and RPL11-related genes (27.37%) were critically involved in UBE2M-depleted HepG2 cells (Figure 2C). Consistently, upregulation of TP53, BAX, PUMA was validated in UBE2M-depleted HepG2 cells by qRT-PCR (Figure 2D). Interestingly, the protein level of p53 upregulation (Figure 3) was higher than the mRNA level of p53, indicating the post-translational effect by UBE2M depletion. In addition, UBE2M depletion increased E-cadherin along with upregulation of apoptosis-related proteins such as p53, Bax and PUMA at mRNA level. In addition, c-Myc, one of the downregulated genes by NGS sequence was validated in HepG2 cells by Western blotting (Figure S1B).

### 3.3. UBE2M Depletion Activates p53 and Maintains Its Stability in HCCs

Consistently with NGS sequence analysis data, UBE2M depletion upregulated p53 at protein levels in p53 wild type HepG2 cells transfected using UBE2M siRNA (Figure 3A). Likewise, UBE2M depletion upregulated p53 in Hep3B cells transfected with p53 and/or UBE2M siRNA (Figure 3B). On the contrary, p53 depletion upregulated UBE2M in HepG2 cells (Figure 3C,D). Conversely, UBE2M overexpression attenuated p53 activation in p53-null type Hep3B cells (Figure 3E) and in HepG2 cells (Figure 3F) by using RGS/His UBE2M plasmids. Furthermore, UBE2M depletion enhanced p53 activation induced by doxorubicin, compared to doxorubicin alone, in HepG2 cells transfected with UBE2M shRNA (Figure 3G). Next, we tested whether or not UBE2M depletion maintained p53 expression levels by evaluating the half-life of p53 in the presence of DNA synthesis inhibitor cycloheximide in HepG2 and HCT116p53+/+ cells. As shown in Figure 3H,I, UBE2M depletion maintained p53 stability in the presence of cycloheximide compared to untreated control in HepG2 and HCT116p53+/+ cells (Figure 3H,I).

### 3.4. Ectopic Expression of UBE2M Enhances Degradation of Exogenous P53 Mediated by MDM2 in HepG2 Cells

**Figure 4:**
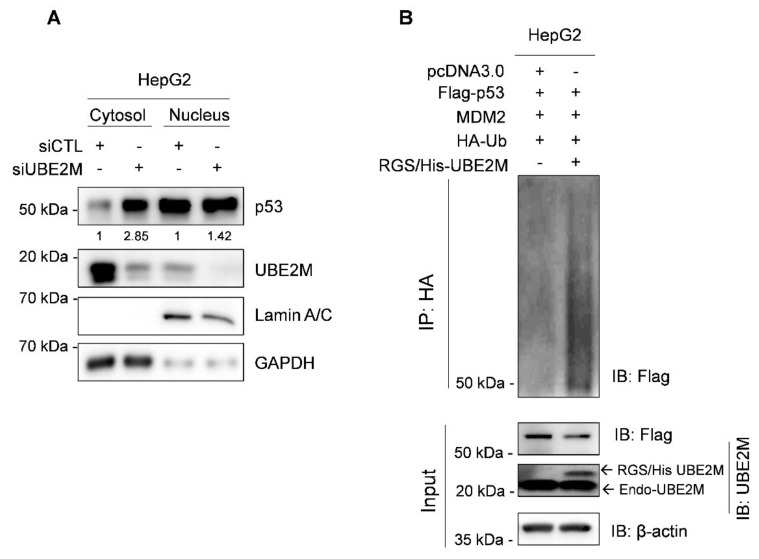
Ectopic expression of UBE2M enhances degradation of exogenous p53 mediated by MDM2 in HepG2 cells. (**A**) Location of UBE2M and p53 in HepG2 cells by fractionation assay. Cytosol and nuclear fractions were isolated and subjected to Western blotting with antibodies of p53, UBE2M and β-actin. (**B**) Effect of UBE2M overexpression on p53 ubiquitination in Hep3B cells. Hep3B cells were co-transfected by plasmids (pcDNA3.0, Flag-p53, MDM2, HA-Ub and RGS/His-UBE2M) and treated with 20 μM MG132 2 h before collecting protein lysates. The lysates were lysed and immunoprecipitated with anti-HA antibody and protein G-agarose beads and immunoblotted with antibodies of Flag, UBE2M and β-actin.

Interestingly, UBE2M is located mainly in the cytosol, while p53 exists in the cytosol and nucleus by fractionation of cytoplasmic and nuclear extracts (Figure 4A). Thus, to test whether UBE2M regulates p53 ubiquitination, an ubiquitination assay was conducted in HepG2 cells transfected with Flag-p53, HA-MDM2, HA-Ub and RGS/His-UBE2M, or UBE2M siRNA followed by MG132 treatment. Herein, the ectopic expression of UBE2M enhanced the ubiquitination of exogenous p53 mediated by MDM2 in HepG2 cells (Figure 4B).

### 3.5. UBE2M Binds to MDM2, but Regulates p53 via Their Crosstalk in HepG2 Cells

**Figure 5:**
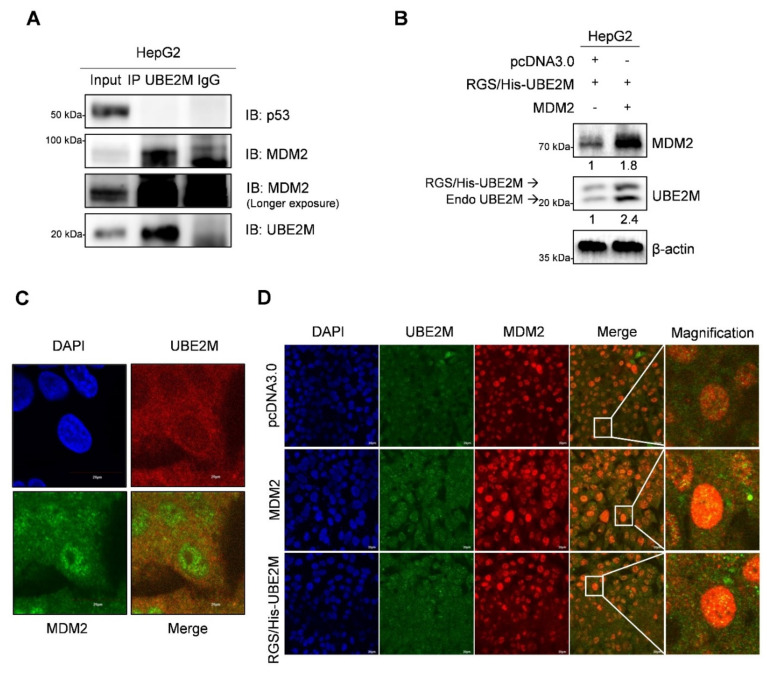
UBE2M binds to MDM2, but regulates p53 via their crosstalk in HepG2 cells. (**A**) UBE2M binds to MDM2, but not to p53, in HepG2 cells. Immunoprecipitation was conducted in HepG2 cells to identify the endogenous interaction between UBE2M and p53, or MDM2 in the presence of MG132. (**B**) UBE2M activates MDM2 in HepG2 cells. HepG2 cells transfected with pcDNA3.0, RGS/His-UBE2M and MDM2 plasmids were lysed and immunoblotted with anti-MDM2, UBE2M and β-actin antibody. (**C**) The colocalization between MDM2 and UBE2M in HepG2 cells at endogenous level by immunofluorescence with ALEXA 488, 596 and DAPI staining. (**D**) The colocalization between MDM2 and UBE2M at exogenous level in HepG2 cells transfected with pcDNA3.0, RGS/His-UBE2M and MDM2 plasmids by immunofluorescence with ALEXA488, 596 and DAPI staining. × 200.

To further examine how UBE2M binds to p53 or MDM2, immunoprecipitation (IP) and immunofluorescence assays were conducted in HepG2 cells. Here, IP reveals that endogenous UBE2M binds to MDM2, but not to p53, in HepG2 cells (Figure 5A). Consistently, UBE2M overexpression enhanced MDM2 expression in HepG2 cells (Figure 5B). Furthermore, the colocalization between UBE2M and MDM2 was observed at an endogenous and exogenous level by immunofluorescence (Figure 5C,D).

### 3.6. UBE2M Depletion Activates p53 and Ribosomal Protein L11 in HepG2 Cells

**Figure 6:**
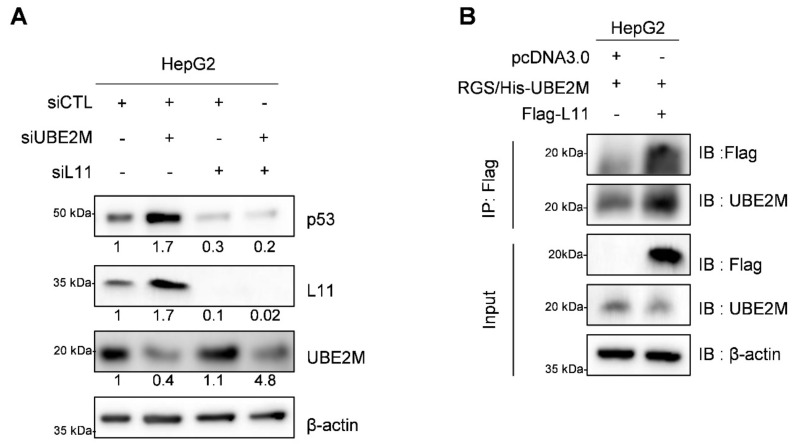
Ribosomal protein L11 is required for p53 activation by UBE2M depletion in HepG2 cells. (**A**) UBE2M depletion activates p53 in the presence of L11. HepG2 cells were co-transfected with control siRNA, UBE2M siRNA and L11 siRNA and were subjected to Western blotting with antibodies of p53, L11, UBE2M and β actin. Total siRNA amount of its transfection mixture was adjusted to 80 nM per one well in 6-well plates. (**B**) UBE2M binds to L11 in HepG2 cells by immunoprecipitation. HepG2 transfected cells with pcDNA3.0, RGS/His-UBE2M and Flag-L11 plasmids were lysed and immunoblotted.

It is well known that ribosomal proteins including L5, L11, L22 and S14 interact with MDM2 to block p53 ubiquitination mediated by MDM2 [33,36,37]. To determine whether UBE2M affected p53 expression through the ribosomal protein L11, Western blotting and immunoprecipitation were conducted in HepG2 cells. Here, L11 knockdown disturbed p53 activation induced by UBE2M depletion, though UBE2M depletion activated p53 and L11 in HepG2 cells (Figure 6A). In contrast, depletion of L5 was not able to block p53 activation. Furthermore, IP revealed that UBE2M binds to L11 in HepG2 cells (Figure 6B). It has been reported that ribosomal proteins bind MDM2 and inhibit p53 degradation.

### 3.7. UBE2M Depletion Retards the Growth of HepG2 Cells Implanted in Balb/c Male Athymic Nude Mouse along with Elevated p53 and Decreased UBE2M Expression by IHC

**Figure 7:**
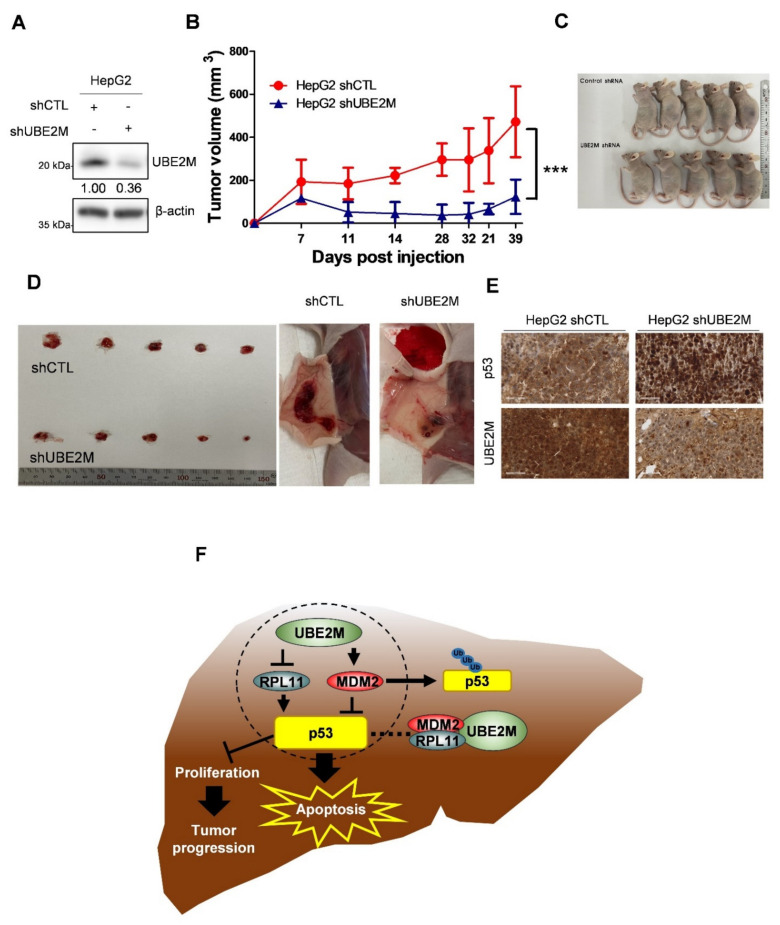
UBE2M knockdown retards the growth of HepG2 cells implanted in Balb/c. (**A**) HepG2 cells transfected with UBE2M shRNA were stabilized and selectively proliferated by puromycin; UBE2M expression level was confirmed by immunoblotting. (**B**) The cells were subcutaneously injected in the flank of Balb/c nude mouse. The tumor size was monitored and measured with the length and width of the tumor for 39 days with a caliper. (**C**,**D**) Thirty-nine days after injection of UBE2M shRNA transfected HepG2 cells, mice and isolated tumors were photographed. (**E**) The tumors were fixed and paraffinized for making blocks and then IHC was conducted with antibodies of p53 and UBE2M. ×40 magnification. (**F**) The schematic diagram of UBE2M signaling associated with MDM2, p53 and RPL11. *** *p* < 0.001 vs. HepG2 shCTL.

An animal study was conducted with Balb/c male athymic nude mice bearing UBE2M shRNA-transfected HepG2 cells to confirm the aforementioned in vitro study. As shown in Figure 7A, UBE2M was successfully depleted in HepG2 cells. In addition, tumor sizes measured by caliper were significantly reduced in the mice group bearing UBE2M-depleted HepG2 cells compared to untreated control group by monitoring for 39 days (Figure 7B), which was confirmed by evaluation of isolated tumors from mice (Figure 7C,D). Furthermore, immunohistochemistry showed that p53 was upregulated while UBE2M was downregulated in tumor sections from the mice implanted by UBE2M d-pleted HepG2 cells compared to untreated control group (Figure 7E).

## 4. Discussion

The underlying molecular mechanism of UBE2M, so called UBC12, for neddylation cascade [19] remains unclear so far, though UBE2M was reported to be overexpressed in hepatocellular carcinoma (HCC) [21] and H1299 lung cancer [22]. In the current study, the molecular mechanism of UBE2M was explored in human hepatocellular carcinoma tissues and cell lines in association with p53/MDM2 and ribosomal protein L11.

UBE2M was overexpressed in HCCs compared to adjacent uncancerous tissues by human tissue array and TCGA analysis, implying the oncogenic potential of UBE2M. Consistently, UBE2M depletion suppressed viability and proliferation in HepG2, Huh7 and Hep3B cells by MTT assay and colony formation assay. In addition, UBE2M depletion cleaved PARP and caspase 3 in HepG2 and Hep3B cells, attenuated the protein expression of EMT molecule Snail and increased E-cadherin, along with upregulation of apoptosis-related proteins such as p53, Bax and PUMA at mRNA level, implying anti-proliferative and apoptotic effects by UBE2M depletion in HCCs.

It is well documented that p53, as an important tumor suppressor, regulates cell-cycle arrest, DNA repair and apoptosis in the cells and also is closely associated with p21, p27, Bax, PUMA and NOXA during the apoptosis process [36,37]. Interestingly, UBE2M depletion activated p53 in HepG2 and HCT116^p53+/+^ cells, demonstrating p53-mediated apoptosis by UBE2M depletion. Similarly, Scott et al. reported that UBE2M knockdown enhances DNA breakages and cellular sensitivity to DNA damaging agents by regulation of CDT1, p21 and claspin [38]. In addition, Zhang et al. [36] reported that UBE2M enhances proliferation and migration in PLC/PRF/5, BEL-7402, SMCC-7721 and L02 cells via activation of β-catenin and cyclin D1.

Interestingly, UBE2M depletion maintained p53 stability in the presence of cycloheximide compared to untreated control in HepG2 and HCT116^p53+/+^ cells, while UBE2M overexpression reduced p53 activation in HepG2 and Hep3B cells. Conversely, p53 knockdown enhanced UBE2M activation in HepG2 cells, though UBE2M is located mainly in the cytosol, while p53 exists in the cytosol and nucleus. However, considering that UBE2M does not bind to p53 in HepG2 cells by immunoprecipitation, the interaction between p53 and UBE2M can be executed via a crosstalk between p53 and UBE2M.

Accumulating evidence reveals that MDM2, the negative regulator of p53, induces p53 degradation and inactivates its tumor suppressing activity through the MDM2–p53 negative feedback loop [39]. Here, UBE2M binds to MDM2 in HepG2 cells by IP; further, the ectopic expression of UBE2M increased MDM2 in HepG2 cells, while the colocalization between MDM2 and UBE2M is observed by immunofluorescence at endogenous and exogenous levels in HepG2 cells. Furthermore, the ectopic expression of UBE2M enhanced the ubiquitination of exogenous p53 mediated by MDM2, while UBE2M depletion reduced p53 degradation induced by MDM2, demonstrating that UBE2M regulates p53 ubiquitination by binding to MDM2.

Previous evidence reveals that ribosomal proteins are critically involved in the MDM2–p53 signaling pathway [32,40]. Hence, suppression of ribosomal biogenesis activates p53 via inhibition of MDM2 E3 ligase [34,41]; further, ribosomal proteins L5, L11 and L23 activate p53 by reducing the MDM2–p53 feedback loop [19,33]. In addition, ribosomal proteins such as RPL5, RPL11 and RPL23 as MDM2 binding partners are known to block the E3 ubiquitin ligase function of MDM2 to promote p53 accumulation [42], indicating the possibility of competitive binding of L11 and MDM2 to UBE2M and ubiquitination or stability of L11 by UBE2M, which should be explored in the future. Herein, UBE2M depletion upregulated ribosomal protein L11 for MDM2 inactivation, while p53 activation was not induced in the absence of RPL11 in HepG2 cells, implying RPL11 is essential in p53 activation.

Additionally, UBE2M depletion reduced HepG2 tumor sizes, increased the expression of p53 and decreased that of UBE2M in tumor tissues isolated from Balb/c male athymic nude mice compared to untreated control, strongly demonstrating the oncogenic potential of UBE2M in association with p53-related signaling.

## 5. Conclusions

Overall, our findings provide a novel insight that UBE2M acts as an oncogene via colocalization or binding with MDM2 or RPL11, despite its working with p53 via crosstalk, not binding, while UBE2M depletion exerts anti-proliferative and apoptotic effect in vitro and in vivo as a target molecule for liver cancer therapy (Figure 7F).

## Data Availability

All the data and materials supporting the conclusions are included in the main paper and the uncropped blots are deposited in the Supplementary Materials; the other data presented in this study are available on request from the corresponding author.

## Supplementary Materials

The following are available online at https://www.mdpi.com/article/10.3390/cancers13194901/s1, Figure S1: Transfection efficiency of UBE2M siRNA and its effect on c-Myc and UBE2M in Hep G2 cells. File S1: uncropped films are shown in a UBE2M raw data file.
