# Supplementary material for: UBE2M Drives Hepatocellular Cancer Progression as a p53 Negative Regulator by Binding to MDM2 and Ribosomal Protein L11
Source: Cancers (Basel). 2021 Sep 29;13(19):4901. doi: 10.3390/cancers13194901 (PMC8507934; doi:10.3390/cancers13194901)
Supplement: Supplementary file 1 [file cancers-13-04901-s001.zip › File S1 uncropped films are shown in a UBE2M raw data file.pptx]

## Slide 1
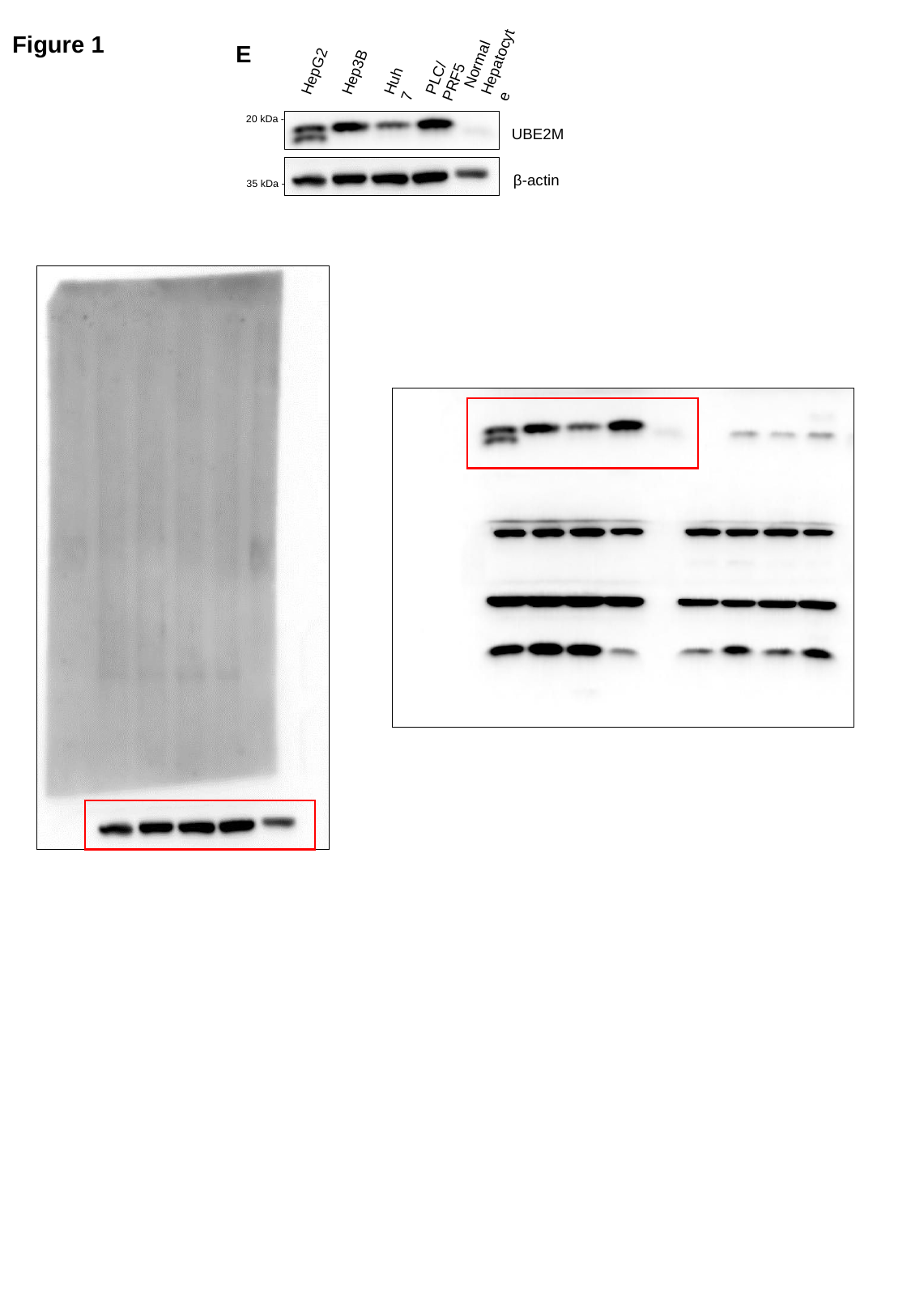

Normal Hepatocyte
HepG2
Hep3B
PLC/PRF5
Figure 1
E
Huh7
20 kDa -
UBE2M
β-actin
35 kDa -

## Slide 2
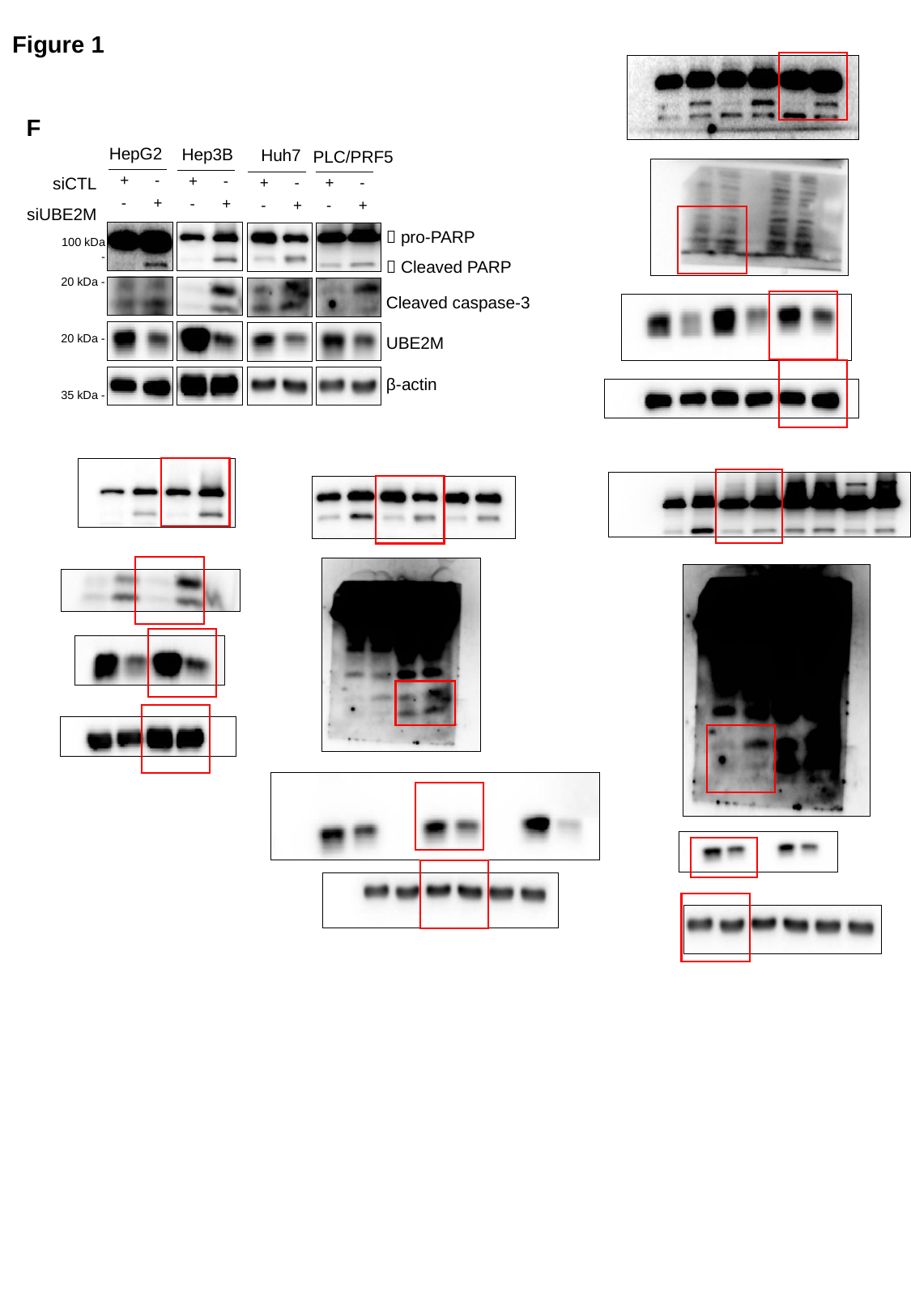

Figure 1
F
HepG2
Hep3B
Huh7
PLC/PRF5
+
-
+
-
+
-
siCTL
+
-
-
+
-
+
-
+
-
+
siUBE2M
 pro-PARP
100 kDa -
20 kDa -
20 kDa -
UBE2M
β-actin
35 kDa -
 Cleaved PARP
Cleaved caspase-3

## Slide 3
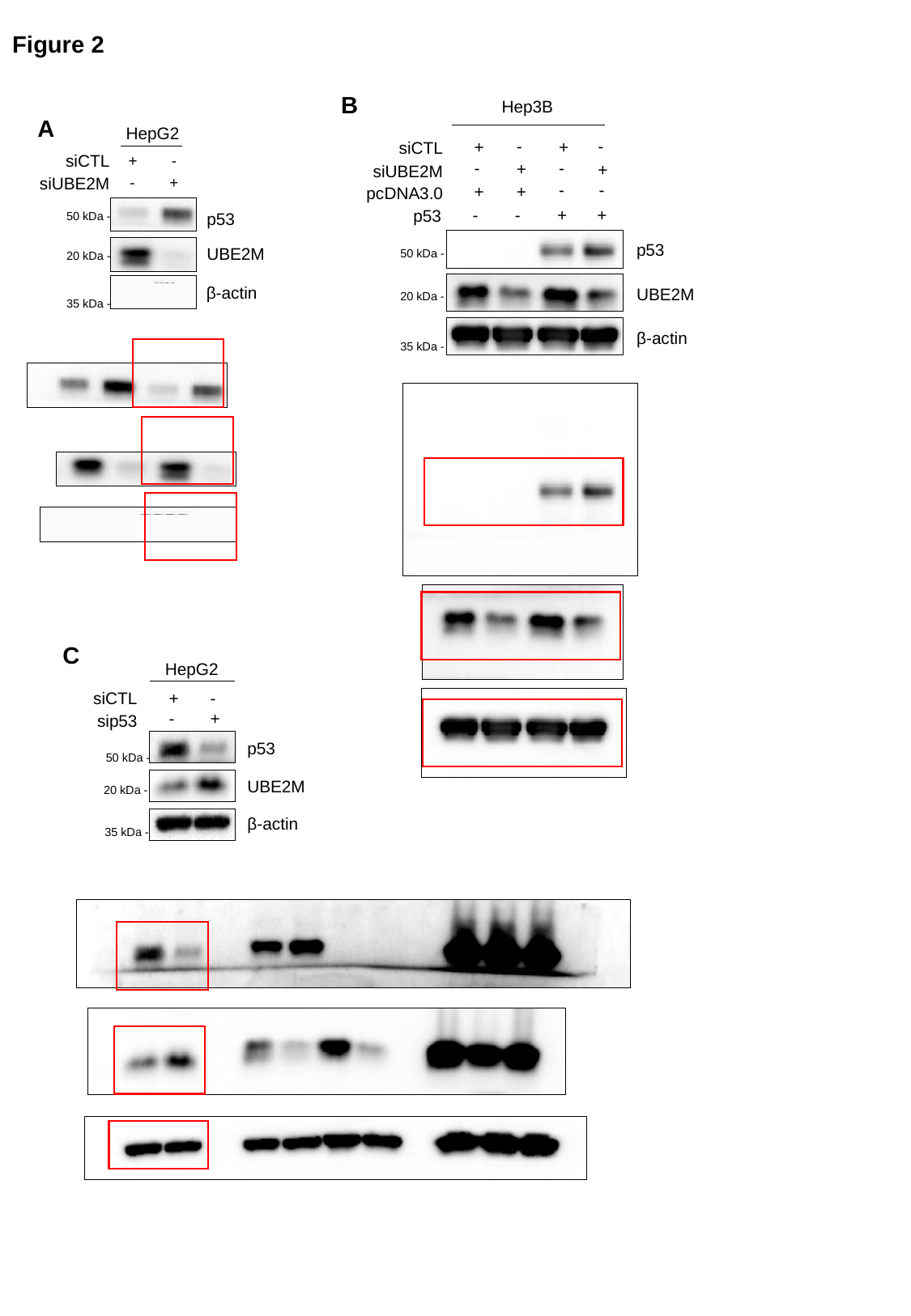

Figure 2
B
Hep3B
A
HepG2
-
-
+
+
siCTL
siCTL
+
-
-
-
+
+
siUBE2M
siUBE2M
-
+
-
-
+
+
pcDNA3.0
-
-
+
+
p53
p53
50 kDa -
p53
UBE2M
50 kDa -
20 kDa -
β-actin
UBE2M
20 kDa -
35 kDa -
β-actin
35 kDa -
C
HepG2
+
-
siCTL
-
+
sip53
p53
50 kDa -
UBE2M
20 kDa -
β-actin
35 kDa -

## Slide 4
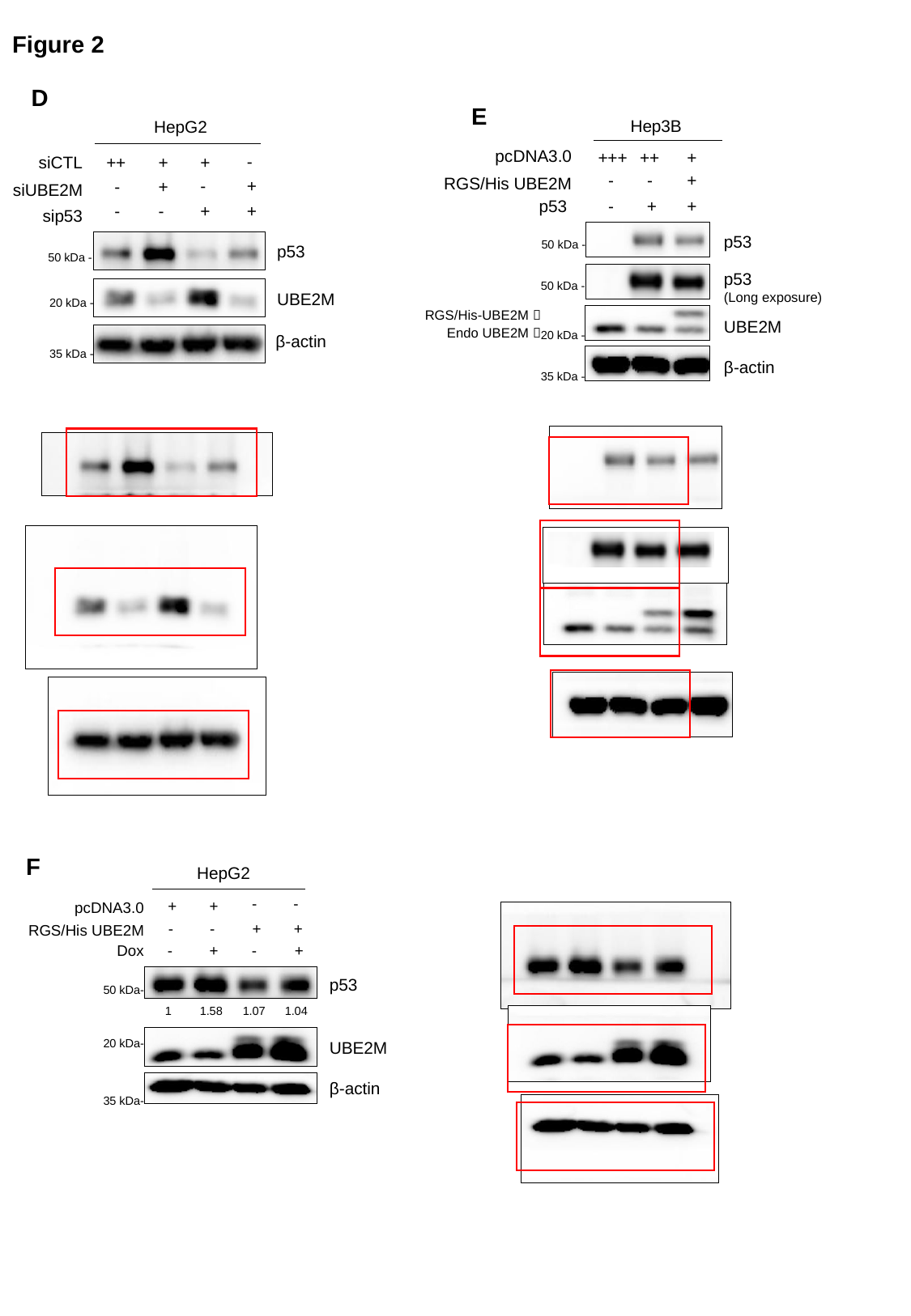

Figure 2
D
E
Hep3B
HepG2
pcDNA3.0
+++
++
+
-
+
+
++
siCTL
-
-
+
RGS/His UBE2M
-
+
-
+
siUBE2M
-
+
+
p53
-
-
+
+
sip53
p53
50 kDa -
p53
50 kDa -
p53
(Long exposure)
50 kDa -
UBE2M
20 kDa -
RGS/His-UBE2M 
UBE2M
Endo UBE2M 
20 kDa -
β-actin
35 kDa -
β-actin
35 kDa -
F
HepG2
-
-
+
+
pcDNA3.0
-
-
+
+
RGS/His UBE2M
Dox
-
+
-
+
p53
50 kDa-
 1
 1.58
 1.07
1.04
 20 kDa-
UBE2M
β-actin
 35 kDa-

## Slide 5
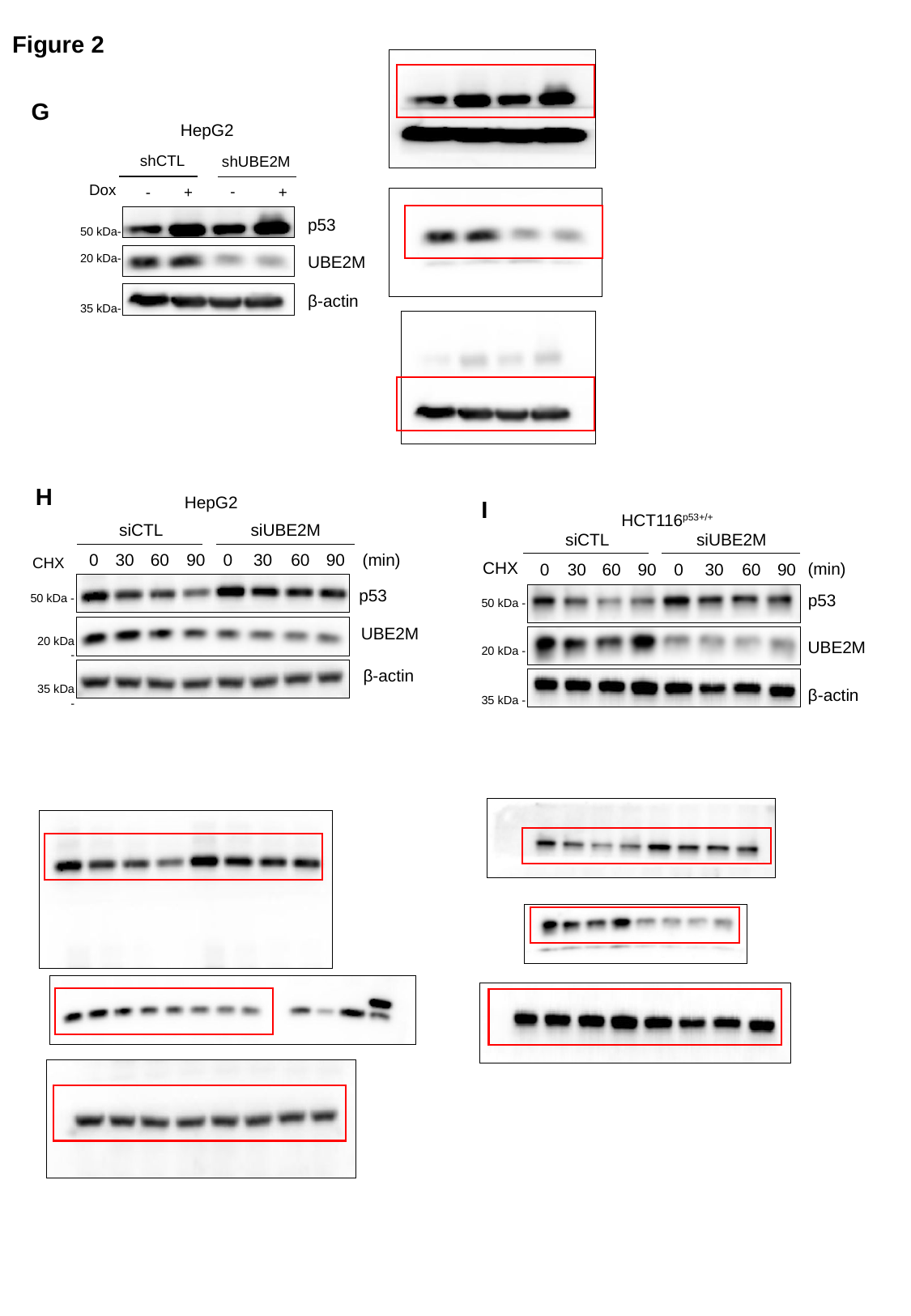

Figure 2
G
HepG2
shCTL
shUBE2M
Dox
-
-
+
+
p53
50 kDa-
 20 kDa-
UBE2M
β-actin
 35 kDa-
H
HepG2
I
HCT116p53+/+
siCTL
siUBE2M
siCTL
siUBE2M
(min)
0
30
60
90
0
30
60
90
CHX
CHX
(min)
0
30
60
90
0
30
60
90
p53
p53
50 kDa -
50 kDa -
UBE2M
20 kDa -
UBE2M
20 kDa -
β-actin
35 kDa -
β-actin
35 kDa -

## Slide 6
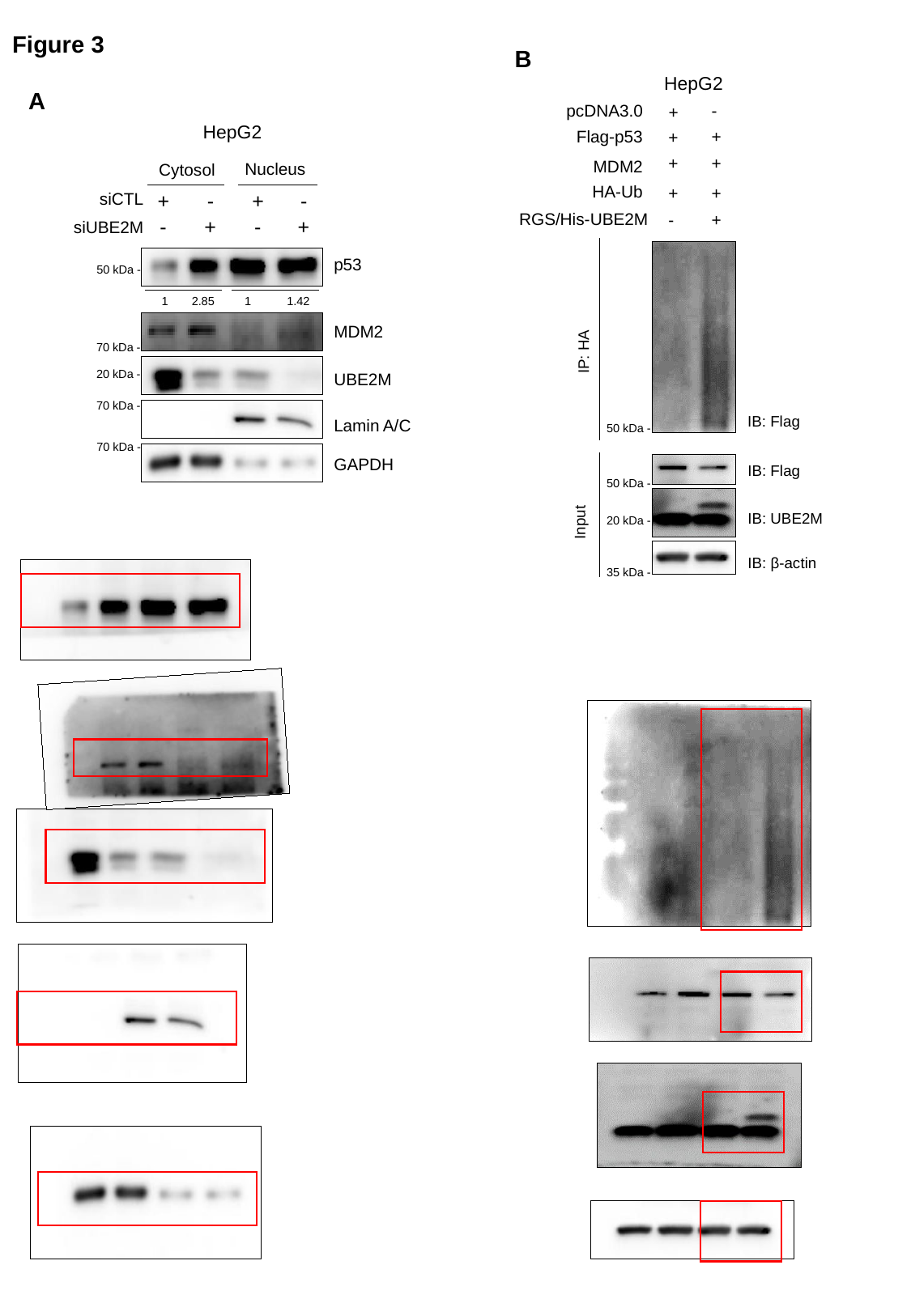

Figure 3
B
HepG2
A
pcDNA3.0
-
+
HepG2
Flag-p53
+
+
+
+
MDM2
Nucleus
Cytosol
HA-Ub
+
+
+
-
+
-
siCTL
RGS/His-UBE2M
-
+
-
+
-
+
siUBE2M
p53
50 kDa -
1
2.85
1
1.42
MDM2
70 kDa -
IP: HA
20 kDa -
UBE2M
70 kDa -
IB: Flag
Lamin A/C
50 kDa -
70 kDa -
GAPDH
IB: Flag
50 kDa -
Input
IB: UBE2M
20 kDa -
IB: β-actin
35 kDa -

## Slide 7
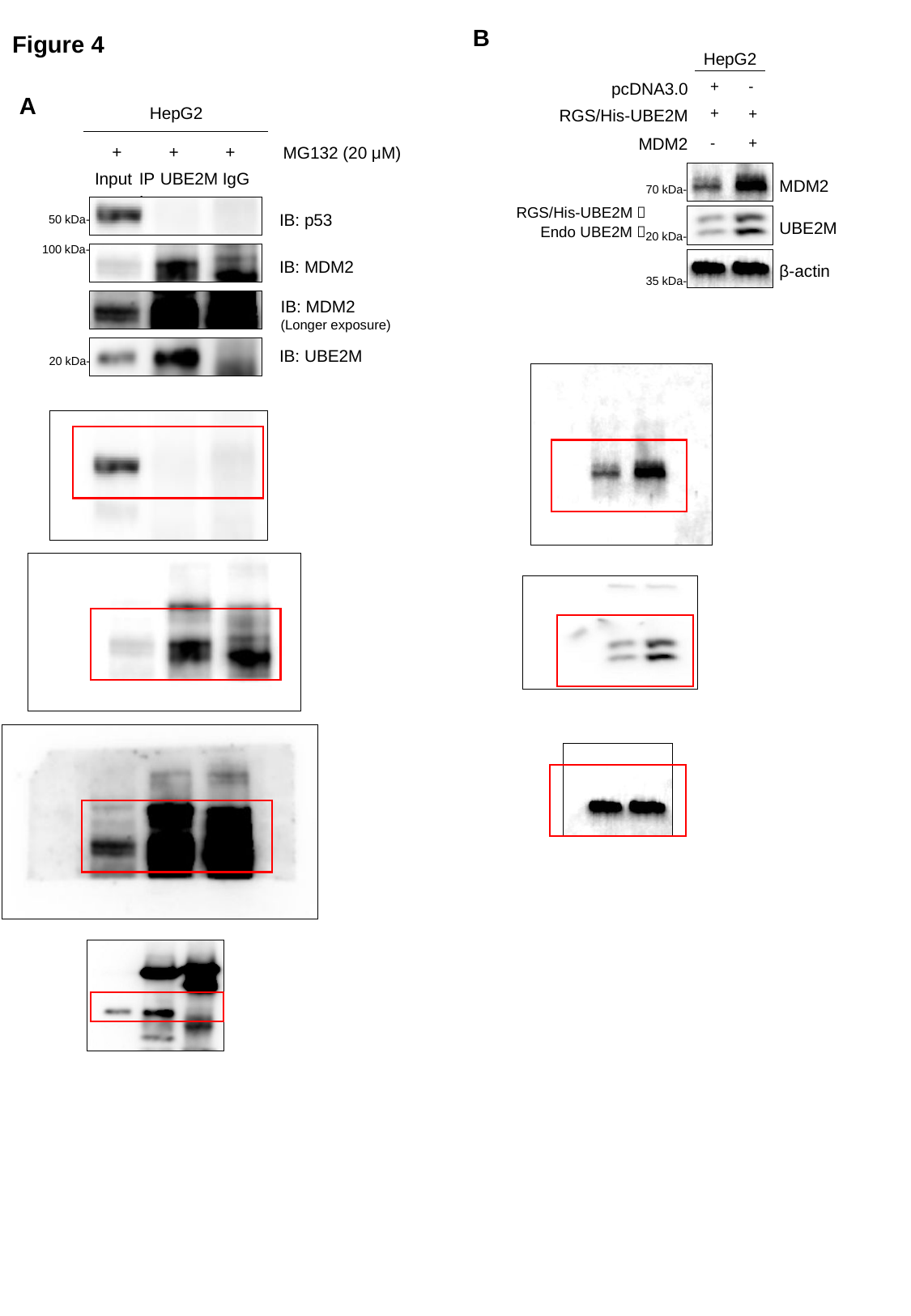

B
Figure 4
HepG2
+
-
pcDNA3.0
A
HepG2
+
+
RGS/His-UBE2M
MDM2
-
+
+
+
+
MG132 (20 μM)
Input
IP:
UBE2M
IgG
MDM2
 70 kDa-
RGS/His-UBE2M 
IB: p53
50 kDa-
UBE2M
Endo UBE2M 
 20 kDa-
 100 kDa-
IB: MDM2
β-actin
 35 kDa-
IB: MDM2
(Longer exposure)
IB: UBE2M
 20 kDa-

## Slide 8
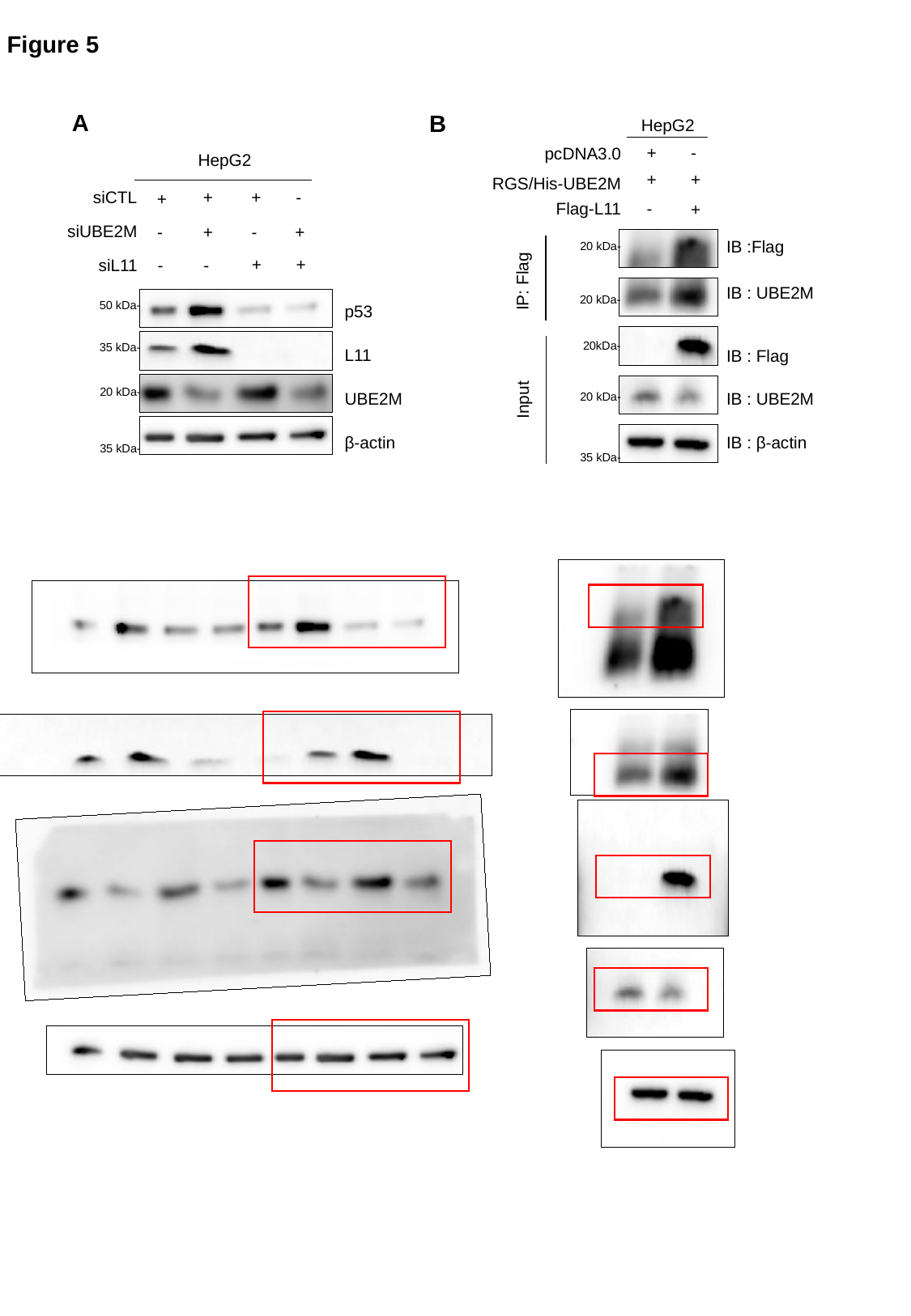

Figure 5
A
B
HepG2
+
-
pcDNA3.0
HepG2
+
+
RGS/His-UBE2M
siCTL
+
+
-
+
Flag-L11
-
+
siUBE2M
-
+
-
+
IB :Flag
 20 kDa-
+
+
-
-
siL11
IP: Flag
IB : UBE2M
 20 kDa-
50 kDa-
p53
20kDa-
 35 kDa-
L11
IB : Flag
 20 kDa-
Input
IB : UBE2M
UBE2M
 20 kDa-
IB : β-actin
β-actin
 35 kDa-
35 kDa-

## Slide 9
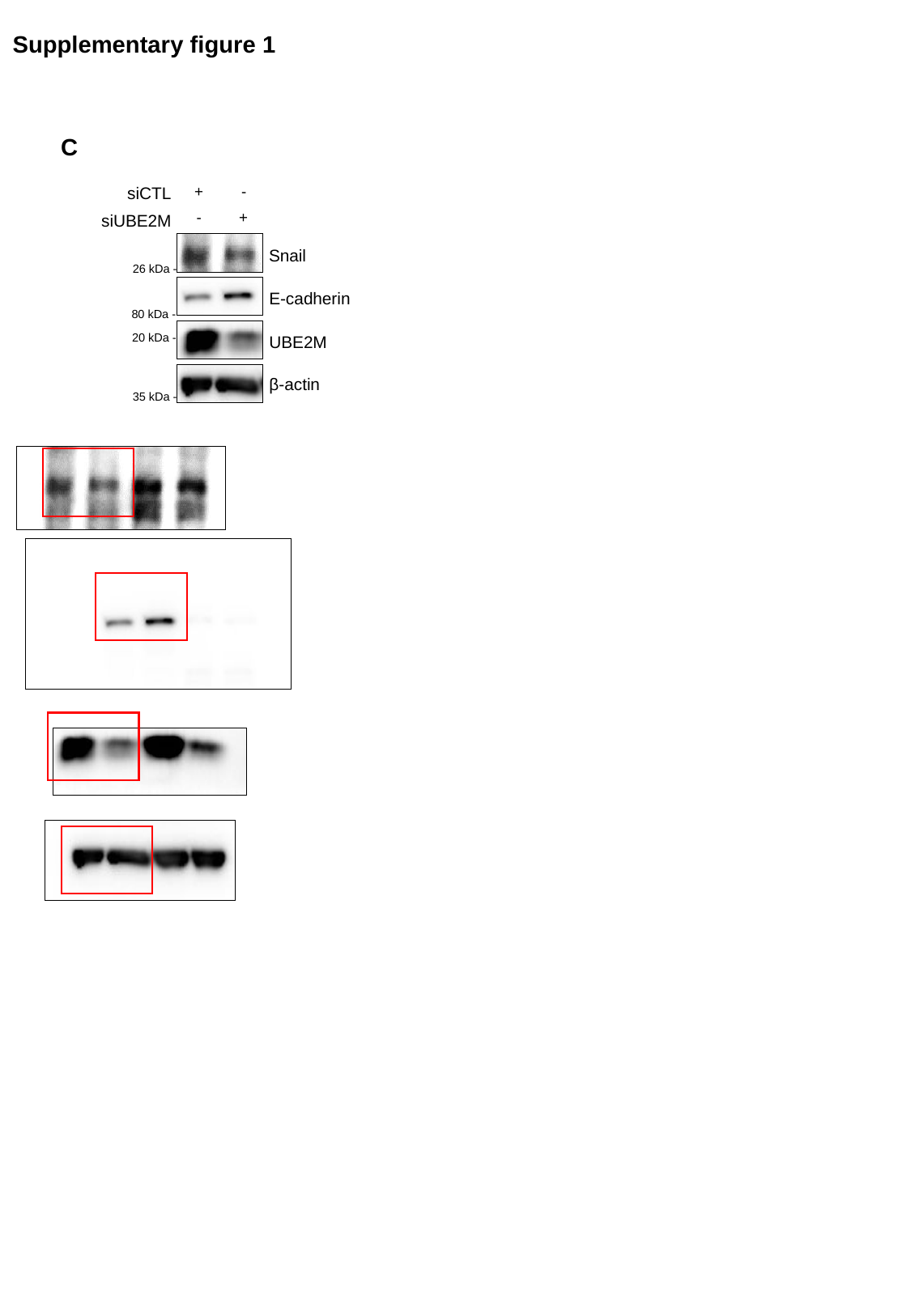

Supplementary figure 1
C
+
-
siCTL
-
+
siUBE2M
Snail
26 kDa -
E-cadherin
80 kDa -
20 kDa -
UBE2M
β-actin
35 kDa -

## Slide 10
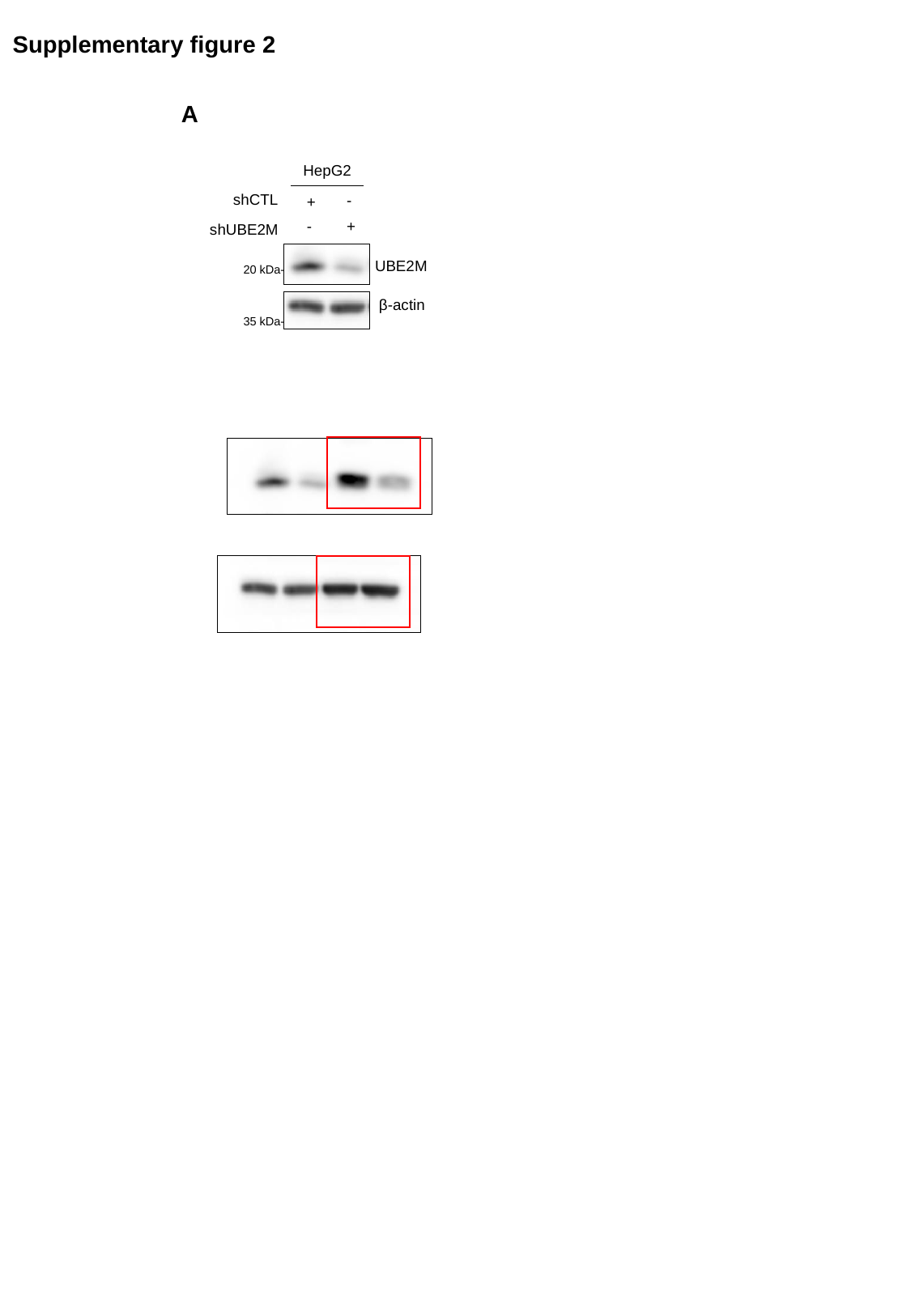

Supplementary figure 2
A
HepG2
shCTL
-
+
-
+
shUBE2M
UBE2M
 20 kDa-
β-actin
 35 kDa-
