# Supplementary figures and images for: UBE2M Drives Hepatocellular Cancer Progression as a p53 Negative Regulator by Binding to MDM2 and Ribosomal Protein L11
Source: Cancers (Basel). 2021 Sep 29;13(19):4901. doi: 10.3390/cancers13194901 (PMC8507934; doi:10.3390/cancers13194901)

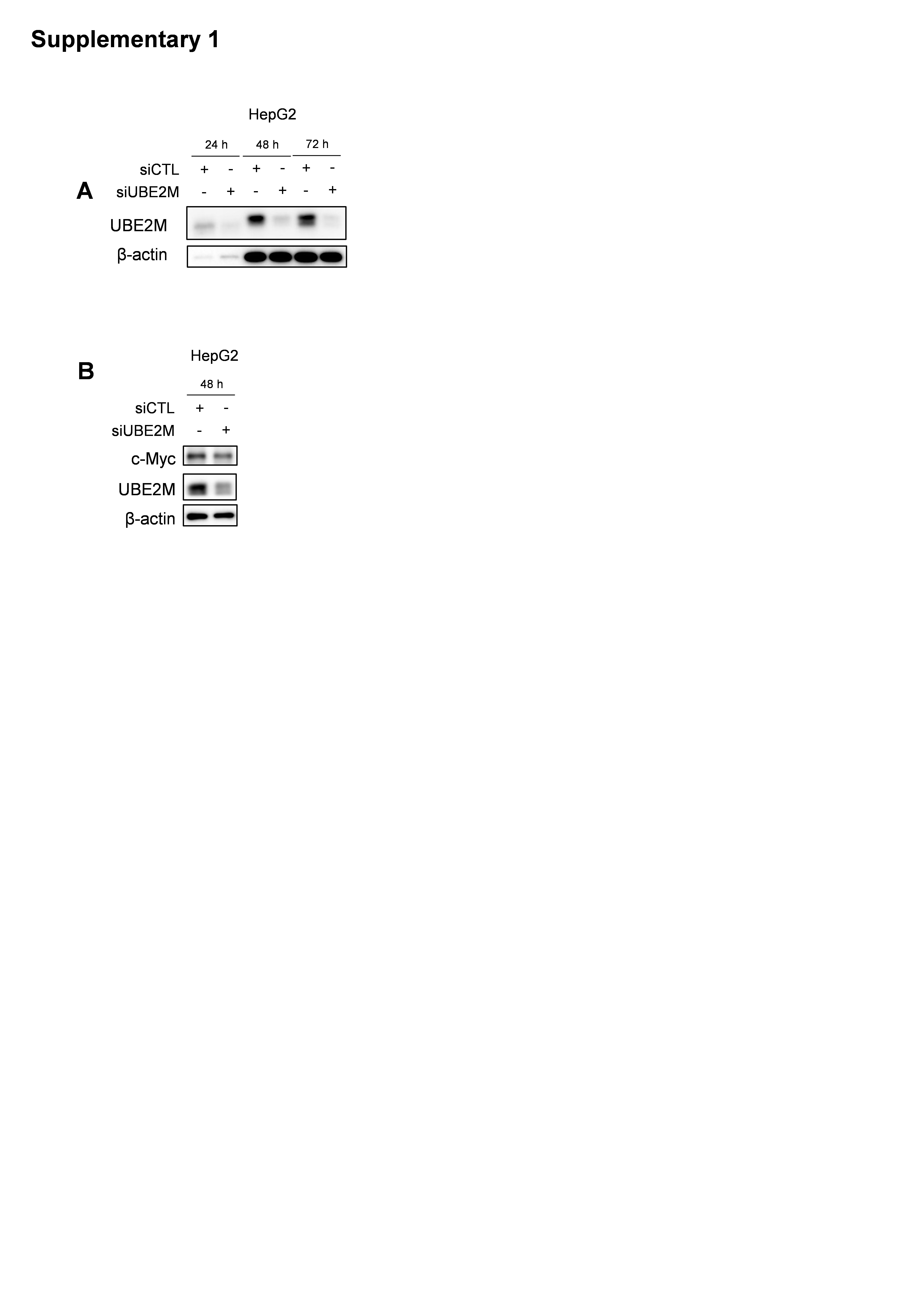

Supplement: Supplementary file 1 [file cancers-13-04901-s001.zip › Figure S1 Transfection efficiency of UBE2M siRNA and its effect on c-Myc and UBE2M in Hep G2 cells.jpg]
